# Hirschsprung's disease in the UK and Ireland: incidence and anomalies

**DOI:** 10.1136/archdischild-2016-311872

**Published:** 2017-03-09

**Authors:** T J Bradnock, M Knight, S Kenny, M Nair, G M Walker

**Affiliations:** 1 Department of Paediatric Surgery, The Royal Hospital for Children, Glasgow, UK; 2 National Perinatal Epidemiology Unit, Nuffield Department of Population Health, University of Oxford, Oxford, UK; 3 Department of Paediatric Surgery, Alder Hey Children's NHS Foundation Trust, Liverpool, UK

**Keywords:** Hirschsprung's disease, Incidence, Associated anomalies, Management

## Abstract

**Objectives:**

To describe clinical characteristics and preoperative management of a national cohort of infants with Hirschsprung's disease (HD).

**Design:**

Population-based cohort study of all live-born infants with HD born in the UK and Ireland from October 2010 to September 2012.

**Setting:**

All 28 paediatric surgical centres in the UK and Ireland.

**Participants:**

305 infants presenting before 6 months of age with histologically proven HD.

**Main outcome measures:**

Incidence, clinical characteristics including gestational age, birth weight, gender, associated anomalies; age and clinical features at presentation; and use of rectal washouts or stoma.

**Results:**

The incidence of HD in the UK and Ireland was 1.8 per 10 000 live births (95% CI 1.5 to 1.9). Male to female ratio was 3.3:1. An associated anomaly was identified in 23% (69), with 15% (47) having a recognisable syndrome. The proportion of infants who presented and were diagnosed in the neonatal period was 91.5% (279) and 83.9% (256), respectively. 23.9% (73) and 44.2% (135) passed meconium within 24 and 48 hours of birth. 81% (246) first presented to a hospital without tertiary paediatric surgical services, necessitating interhospital transfer. Initial colonic decompression was by rectal washouts in 86.2% (263) and by defunctioning stoma in 12.8% (39). Subsequently, 27.4% (72) of infants failed management with rectal washouts and required a delayed stoma, resulting in 36.4% (111) of infants having a stoma.

**Conclusions:**

In this population-based cohort, presentation outside the neonatal period was rare. Nearly half of the infants with HD passed meconium within 48 hours of birth and over one third were managed with a stoma.

**What is already known on this topic?**

- The estimated incidence of Hirschsprung's disease (HD) is approximately 1 in 5000 live births and more than 90% of cases present in the neonatal period.
- Most information on the epidemiology and early management of HD comes from retrospective case series, voluntary reporting surveys and surveys of intended practice.
- There is little published information on interactions between infants with HD and healthcare services prior to a diagnosis of HD being established.

**What this study adds?**

- A quarter of infants with Hirschsprung's disease (HD) pass meconium within 24 hours of birth and nearly half within 48 hours of birth, limiting the validity of ‘timing of first meconium’ as a screening question for HD.
- One in three infants with HD is discharged home after birth, prior to diagnosis, placing a heavy burden on primary care to ensure timely referral.
- More than a third of infants with HD receive a stoma prior to definitive surgery—a higher rate than reported internationally and in contrast to surgeons reported intent.

## Introduction

Hirschsprung's disease (HD) is characterised by the absence of intrinsic parasympathetic ganglia (aganglionosis) in the distal bowel, resulting in functional intestinal obstruction. Patients classically present during the neonatal period or early infancy.^1^ Initial supportive management is followed by definitive surgery, involving resection of the aganglionic colon below the histological ‘transition zone’ (TZ) and anastomosis of ganglionic bowel to the anorectum.^2–5^

Worldwide, the estimated incidence of HD is approximately 1 in 5000 live births,^6^ ^7^ but there are no nationally representative data to provide an accurate picture of the incidence, demographics and mode of presentation of HD in the UK and Ireland. Available regional data regarding incidence and associated anomalies are limited by a restricted study population, data collection over prolonged periods of time^8^ or include cases identified more than 50 years ago.^9^ ^10^

Following confirmation of HD, initial management aims to maintain colonic decompression. Most paediatric surgeons advocate rectal washouts to achieve this, aiming to perform a primary pull-through,^11^ removing the aganglionic bowel without a preceding stoma.^12^ Some infants fail to decompress adequately with rectal washouts and require a stoma to achieve satisfactory colonic decompression, and some are deemed unsuitable from the outset. The majority of previous studies examining the early management of HD comprise retrospective case series,^13^ ^14^ voluntary reporting surveys with variable methods of case ascertainment,^1^ ^7^ ^15^ surveys of intended practice^11^ ^16^ ^17^ or meta-analyses and systematic reviews of retrospective case series.^18–20^ To date, there are very few prospective, population-based observational studies of HD anywhere in the world^1^ ^7^ and none that provide representative data in a cohort of children born during a short time period.

The aims of this study were to describe the incidence, clinical characteristics and management prior to definitive surgery in a national cohort of infants with HD in the UK and Ireland.

## Methods

All live-born infants, up to 6 months of age, diagnosed with HD (defined as an absence of ganglia in the enteric nervous system of the distal bowel), between the 1 October 2010 and 30 September 2012 were eligible for inclusion in the study. Cases were identified using the British Association of Paediatric Surgeons Congenital Anomalies Surveillance System.^21^ Each month, we sent a reporting card to nominated reporting clinicians in all 28 paediatric surgical units in the UK and Ireland, requesting the number of infants diagnosed with HD in their unit that month. In response to a report indicating a new case of HD, we sent a data collection form requesting further details including basic demographic data, age and clinical features at presentation, associated anomalies, early management prior to definitive surgery and site of histopathological TZ. Up to five reminders were sent if the data collection form was not returned.

Duplicate reports were eliminated by comparing hospital of birth, gestation at birth and date of notification and follow-up with the reporting clinicians.

### Statistical analyses

Descriptive statistics were used to describe the basic demographics, associated anomalies and mode of presentation. We calculated the rate of HD with 95% CIs among live-born infants by using the denominator of total reported live births in England and Wales,^22–24^ Scotland,^25^ Northern Ireland^26^ and the Ireland^27^ during the study period, 1 October 2010 to 30 September 2012. All statistical analyses were performed using STATA V.14.

## Results

**Figure 1:**
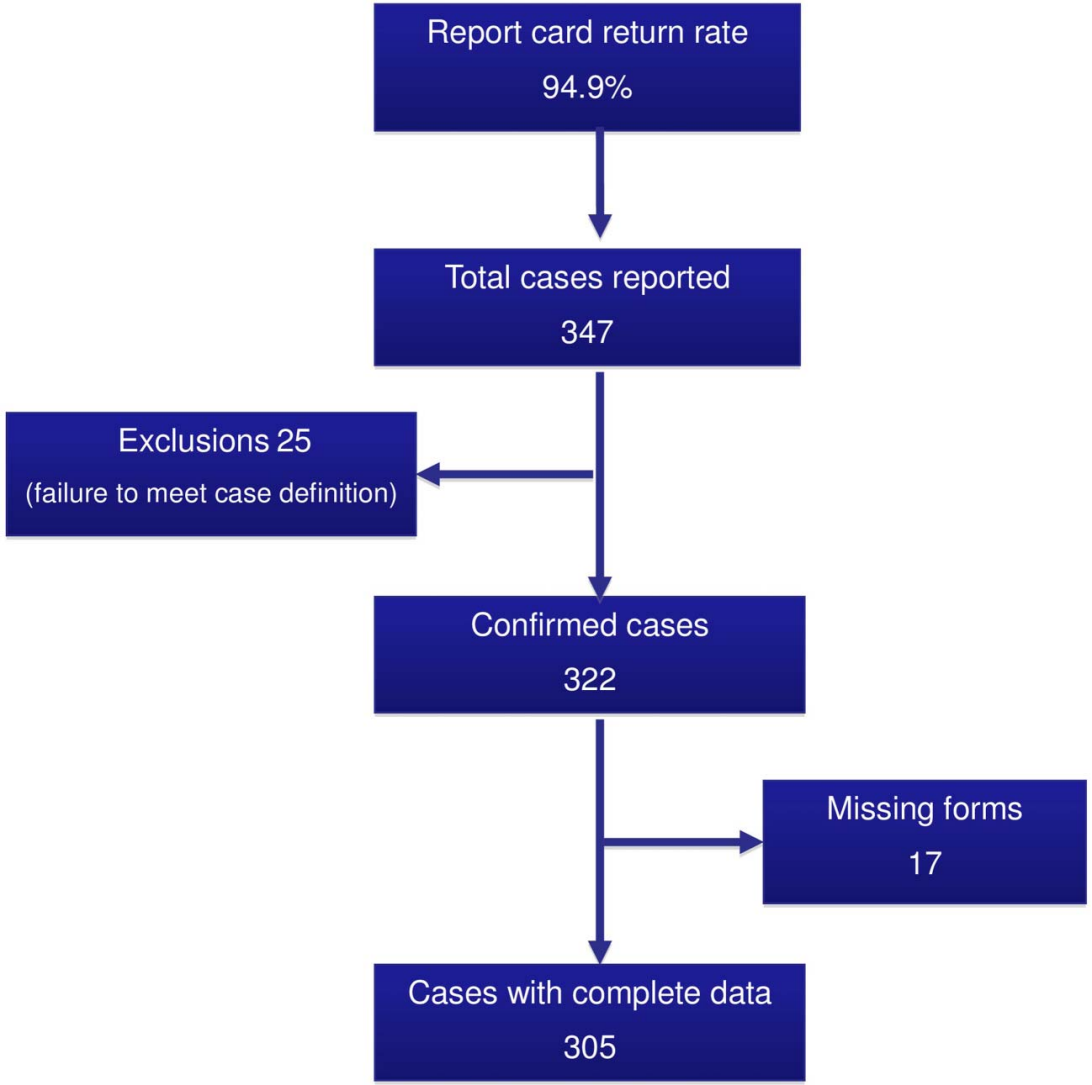
Case ascertainment and data collection.

Between 1 October 2010 and 30 September 2012, 305 infants with HD were identified in the UK and Ireland. Figure 1 summarises case ascertainment, exclusions and data collection for the study.

### Incidence

Over the same period, there were 1 729 854 live births in the UK and Ireland.^22–27^ The incidence of HD in the UK and Ireland was thus estimated as 1.8 per 10 000 live births (95% CI 1.5 to 1.9).

### Basic demographics, associated anomalies and mode of presentation

The basic demographics, associated anomalies and presenting features for the cohort are summarised in table 1. Median gestational age was 39 weeks (range 28–42). Thirty-eight infants (12%) were born prematurely (defined as before 37 weeks completed gestation) and 19 infants (6.2%) were born at less than 35 weeks gestation. Median birth weight was 3400 g (range 1000–4900 g). Median age at presentation and diagnosis were 2 (range 1–159) and 9 days (range 1–177), respectively. Median time from presentation to diagnosis was 5 days (range 0–176), and 256 infants (84%) were diagnosed during the neonatal period.

**Table 1:** Demographics, associated anomalies and presenting features in 305 infants with Hirschsprung's disease

Characteristic	
Gestational age (weeks)	
≥37 weeks	86.6 (264)
<37 weeks	12.5 (38)
Missing	1 (3)
Gender	
Male	76.7 (234)
Female	23.0 (70)
Missing	0.3 (1)
Ethnicity	
White	85.3 (260)
Non-white	13.4 (41)
Missing	1.3 (4)
Birth weight (g)	
≥2500	85.3 (260)
<2500	11.2 (34)
Missing	3.6 (11)
Positive family history	
Yes	8.5 (26)
No	90.2 (275)
Missing	1.3 (4)
Associated anomalies	22.6 (69)
Syndromic association	15.4 (47)
Isolated additional anomaly	7.2 (22)
Missing	0.7 (2)
Features at presentation	
Abdominal distension	92.8 (283)
Bilious vomiting	66.9 (204)
Non-bilious vomiting	19.3 (59)
Not opening bowels	11.2 (34)
Poor feeding	9.2 (28)
Suspected enterocolitis	9.2 (28)
Perforation	1.6 (5)
Any other presentation	4.9 (15)
Timing of 1st meconium	
<24 hours	23.9 (73)
24–48 hours	20.3 (62)
>48 hours	38.4 (117)
No spontaneous	4.6 (14)
Missing	12.8 (39)

Note: Figures are percentage and (frequency or range/IQR).

Overall, the HD cohort included 3.3 times more male than female infants (male 234 vs female 70) and 26 (9%) infants had a positive family history. An associated anomaly was identified in 69 infants (23%), with 47 (15%) of these having a recognisable syndromic association, including Down syndrome (27, 9%), Mowat-Wilson syndrome (5, 2%), congenital central hypoventilation syndrome (3, 1%) or Bardet-Biedl syndrome (2, 1%). An associated cardiac anomaly was identified in 29 infants (10%), which occurred in the context of an underlying predisposition syndrome in 22 out of 47 infants (47%) and in 7 out of 258 non-syndromic infants (3%). An associated urological anomaly was identified in 11 infants (4%), which occurred in the context of an underlying predisposition syndrome in 4 out of 47 infants (9%) and in 7 out of 258 non-syndromic infants (3%).

### Presentation

Abdominal distension and bilious vomiting were the most common clinical features at presentation (table 1). In combination, these two features were present in 188 infants (61.6%). Meconium was passed spontaneously within 24 hours of birth in 73 infants (24%) and within 48 hours in 135 infants (44%). The ‘classic triad’ of bilious vomiting, abdominal distension and delayed passage of meconium was evident in only 80 infants (26.2%). The first presentation with symptoms and signs associated with HD occurred in a hospital without tertiary paediatric surgical services in 246 (81%) infants, necessitating transfer to a paediatric surgical centre. One hundred and three (34%) infants presented from home.

### Maintenance of colonic decompression

**Figure 2:**
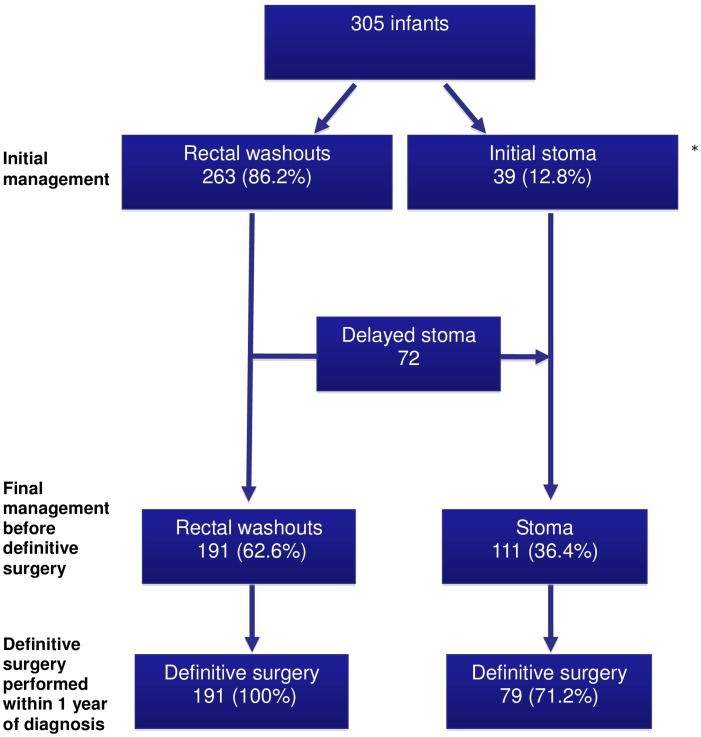
Maintenance of colonic decompression and the proportion of infants undergoing definitive surgery at 1 year after diagnosis. *Three infants not included—one died and two had a primary pull-through without preceding stoma or rectal washouts.

The initial method for colonic decompression (rectal washout vs stoma), the final management prior to definitive surgery and the proportion of infants in each group undergoing definitive surgery within 1 year of diagnosis are summarised in figure 2. Rectal washouts were used in 263 (86%) infants, and 179 (68%) infants had this treatment at home. A stoma was performed in 39 (13%) infants without an initial trial of rectal washouts. One infant died and two underwent a primary pull-through without preceding stoma or rectal washout. A further 72 infants subsequently underwent stoma formation, having initially been managed with rectal washouts. In total, 111 (36%) infants received a stoma prior to definitive surgery, at a median age of 13 days (range 1–367). Indications for stoma formation included failure to decompress with rectal washouts (42), emergency laparotomy (25), suspected long-segment disease (16), enterocolitis (10), consultant preference for a staged approach in all cases (7), comorbidity (7), failure to manage rectal washouts (6) and delayed presentation (1). Following confirmed histological diagnosis, 243 (80%) infants were allowed home before definitive surgery.

### Length of aganglionosis

Definitive surgery was carried out in 270 (89%) infants within 1 year of diagnosis, establishing the length of aganglionosis. The pathological TZ was rectosigmoid in 198 (73.3%), long segment (proximal to the sigmoid colon) in 60 (22.2%), total colonic in 8 (3.0%) and unknown in 4 (1.5%).

### Mortality

Nine infants (3%) died without undergoing definitive surgery. Seven of these infants had a stoma in situ at the time of death and seven had a syndromic association. The median age at death was 85 days (range 11–381). The cause of death was attributed to an underlying cardiac anomaly in five infants, sepsis in two infants, multiple comorbidities prompting withdrawal of treatment in one infant and was unclear in one infant.

## Discussion

This study provides robust, population-level data for the UK and Ireland, with data collected directly from local surgeons, rather than ‘second hand’ from administrative databases. The population-based nature, together with high rates of case ascertainment and data accrual achieved, allow an accurate estimate of the incidence, clinical characteristics and current preoperative management of infants with HD in the UK and Ireland.

To enable future comparative, age-matched analysis of long-term functional outcome, we limited our cohort to infants diagnosed before 6 months of age. Contemporary population-based studies confirm that around 90% of infants with HD are diagnosed within the neonatal period^1^ and late-presenting cases are rare.^28^ While it is likely that a small number of cases presenting after 6 months of age will not have been captured, we feel that this will have little impact on incidence calculations and that our cohort is representative of the vast majority of children with HD in the UK and Ireland.

For a condition with a complex pattern of polygenic inheritance, characterised by variable sex-dependent penetrance of the most common known genetic mutations,^29^ the incidence of HD worldwide appears consistent with the caveat that most studies originate from the Caucasian diaspora.^1^ ^6^ ^7^ In the UK and Ireland, two small, non-population-based studies from the 1980s and 1960s, respectively, estimated the incidence of HD to be 1 in 4500 live births^9^ and 1 in 2000–10 000 live births,^10^ but both studies were limited, either by a protracted study period^9^ or through collection of cases from a wide geographical area, with a poorly defined study population.^10^ The incidence of 1.8 per 10 000 live births calculated from our study is comparable to the incidence of 1.63 per 10 000 live births (95% CI 1.33 to 1.98) identified in the North of England using the Northern Congenital Abnormality Survey between 1990 and 2008.^8^ A recent European Surveillance of Congenital Anomalies study covering 31% of the European birth population between 1980 and 2009^30^ found a total prevalence of 1.09 per 10 000 live births (95% CI 1.03 to 1.15) with marked regional variation in prevalence. The authors concede that this may result from differences in case definition and heterogeneity in regional reporting rates, which may also account for the lower than expected incidence compared with the majority of published series.^1^ ^6^ ^7^

In our cohort, 12% of infants with HD were born prematurely; nearly twice the overall rate of preterm birth for England and Wales (7.3%)^31^ and Scotland (5.9%)^32^ during the study period. A recent systematic review^19^ reported a 6% rate of preterm birth in HD and suggested a rising prevalence. Baxter and Bhatia^33^ suggest that the observed rise in prevalence of prematurity in HD may be attributable to a rising incidence of prematurity overall, but since the population incidence of prematurity has remained at 7.3% for England and Wales between 2009 and 2012,^31^ and has fallen from 6.7% in 2003/2004 to 5.9% in 2011/2012 in Scotland,^32^ other factors should be considered. Population-based birth defect surveillance systems have previously identified an association between other major birth defects and preterm birth.^34^ Far from being rare in infants with HD, there is growing evidence of an association between HD and preterm birth, and future work should seek to clarify this potential relationship.

In our cohort, more than one in five infants had an associated anomaly, with a syndromic association in 15% and an isolated additional anomaly in 7%. Overall, 1 in 11 infants had Down syndrome and this group accounted for 57.4% of infants with a predisposing syndrome. Infants with Down syndrome are estimated to have 40 times increased risk of HD.^35^ This was confirmed by a recent meta-analysis of more than 16 000 infants with HD, which found a 7.3% rate of Down syndrome, compared with an expected rate of 0.15%–0.17% in the general population.^20^ The finding that HD occurred as an isolated trait in 77.4% of infants in this series is in agreement with previous studies.^29^

Between 94%^36^ and 98.5%^37^ of normal-term infants pass meconium within 24 hours of delivery and the remainder by 48 hours.^37^ Previously, it has been suggested that less than 10% of infants with HD pass meconium within 24 hours of birth.^38^ In this study, a quarter of infants with HD passed meconium within 24 hours of birth and nearly half of infants within 48 hours of birth. Two recent studies^1^ ^13^ also reported that around 40% of infants with HD, including preterm infants,^13^ passed meconium within 48 hours of birth. Current National Institute for Health and Care Excellence (NICE) guidance on ‘Constipation in Children and Young People’ incorporates ‘delayed passage of meconium beyond 48 hours in term infants’ as a red flag for urgent specialist referral to exclude HD.^39^ Our findings suggest that the apparently timely passage of meconium should not lead the clinician to refute a diagnosis of HD, particularly in the presence of other ‘red flag’ symptoms such as abdominal distension.

Although paediatric surgeons are familiar with the management of HD, our findings indicate that the majority of these infants initially present to other primary or secondary healthcare services in the UK and Ireland. One third of infants in this cohort were discharged home after birth, prior to HD being suspected or diagnosed and over 80% first presented to a hospital without tertiary paediatric surgical services and required transfer to a paediatric surgical centre. As the majority of these infants presented initially to non-surgical specialities, these data highlight the importance of regional networking, including robust referral pathways to tertiary care services.

Surveys of practice have demonstrated clear changes in surgeons' preference for the initial colonic decompression of infants with HD, with a move away from a staged approach and increased popularity of the primary pull-through.^11^ ^15^ ^16^ In a recent survey of *intended* practice of UK paediatric surgeons, only 15% would aim to perform a stoma prior to definitive surgery.^11^ In the presented cohort, a defunctioning stoma was *actually* performed in more than one third of infants, with 13% receiving a stoma without any attempts at rectal washouts, and a further 27% of infants initially managed with rectal washouts receiving a stoma, after the washouts failed to achieve adequate decompression. These findings provide robust information that can be used to counsel parents of infants with HD. Limited data exist to explain the higher than expected stoma rates observed in clinical practice compared with surveys of *intended* practice. In this cohort, the indications for stoma formation were mostly due to clinical concerns, rather than a consultant preference for this approach. Future work will be directed at identifying factors that increase the likelihood of stoma formation.

## Conclusions

This study identified a national cohort of infants with HD in the UK and Ireland. The data provide a robust estimate of the incidence, clinical characteristics and associated anomalies of HD in the UK and Ireland. We report national outcome data for clinical markers such as time from presentation to diagnosis and stoma utilisation rates that can be used as a benchmark against which practice and outcomes in single centres can be compared and future changes in service provision measured. The data provide further evidence to challenge some of the previously held dogmas in HD, including the prevalence of preterm birth and the validity of timing of first meconium as a screening question for HD. There appears to be an increased use of home rectal irrigations and high stoma rates, both of which rely on adequate support services in the community. Furthermore, one in three infants with HD is discharged home after birth, prior to a diagnosis of HD, placing a heavy burden of responsibility on health visitors and general practitioners to ensure timely referral of these infants.
